# Meeting report of Ctenopalooza: the first international meeting of ctenophorologists

**DOI:** 10.1186/s13227-016-0057-3

**Published:** 2016-08-25

**Authors:** Joseph F. Ryan, Christine E. Schnitzler, Sidney L. Tamm

**Affiliations:** 1 Whitney Laboratory for Marine Bioscience, University of Florida, St Augustine, FL 32080 USA; 2 Department of Biology, University of Florida, Gainesville, FL 32611 USA; 3 National Human Genome Research Institute, National Institutes of Health, Bethesda, MD 20892 USA; 4 Bell Center, Marine Biological Laboratory, Woods Hole, MA 02543 USA

**Keywords:** Ctenophora, Ctenophore, Ctenopalooza

## Abstract

Here we present a report on Ctenopalooza: A meeting of ctenophorologists held at the Whitney Laboratory for Marine Bioscience in St. Augustine, FL, USA, on March 14–15, 2016. In this report, we provide a summary of each of the sessions that occurred during this two-day meeting, which touched on most of the relevant areas of ctenophore biology. The report includes some major themes regarding the future of ctenophore research that emerged during Ctenopalooza. More information can be found at the meeting Web site: http://ctenopalooza.whitney.ufl.edu.

## Introduction

**Fig. 1:**
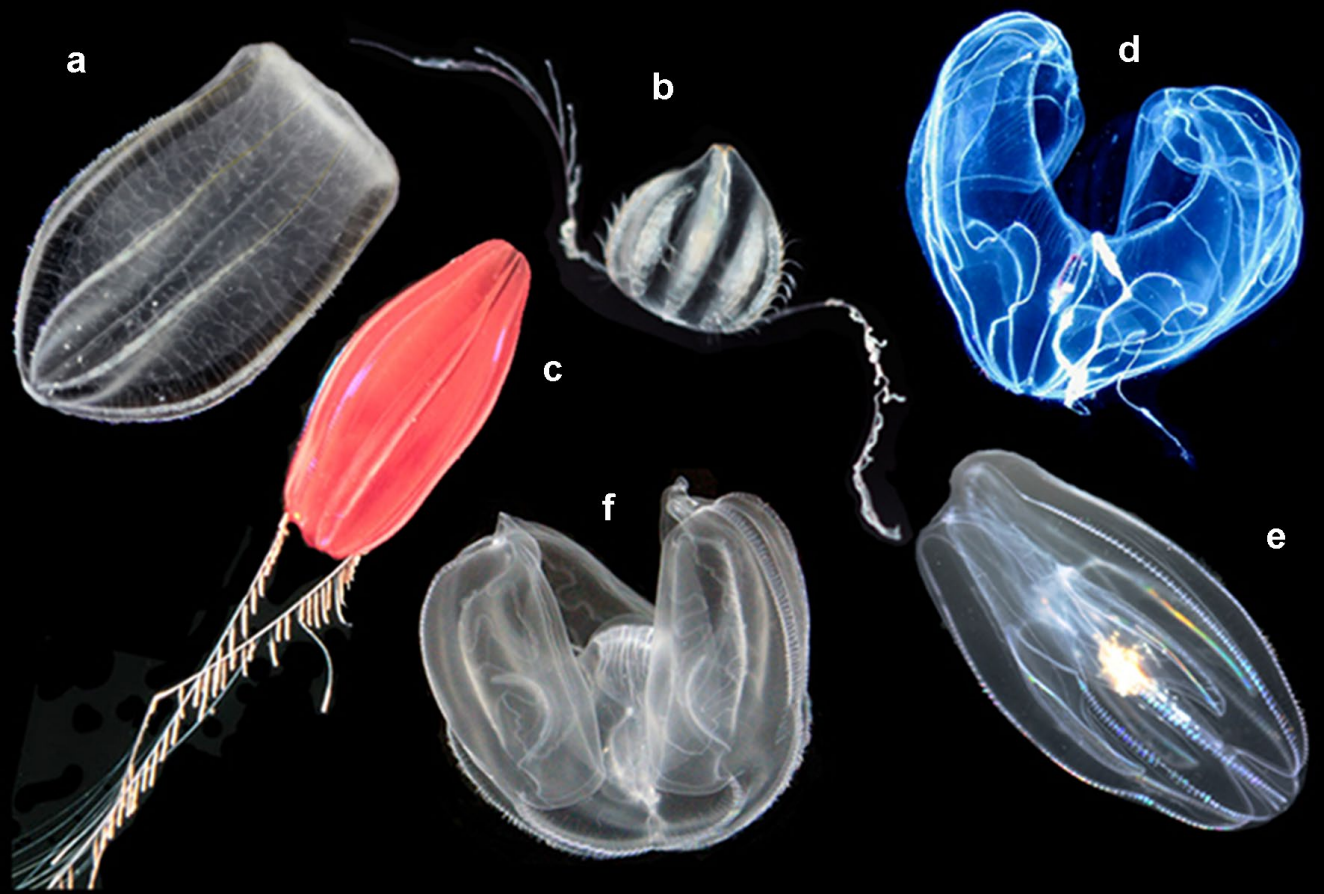
Pelagic ctenophores—(*a*) *Beroe ovata*, (*b*) *Euplokamis* sp., (*c*) *Nepheloctena* sp., (*d*) *Bathocyroe fosteri*, (*e*) *Mnemiopsis leidyi*, and (*f*) *Ocyropsis* sp. Photograph credits: (*a*, *b*, *e*, *f*—Joseph F. Ryan) (*c*—R. Griswold, National Oceanic and Atmospheric Administration) (*d*—Marsh Youngbluth, National Oceanic and Atmospheric Administration). Images *a*, *b*, *e*, *f* are licensed under Creative Commons (CC-BY); images (*c*, *d*) are in the public domain

Ctenophores, also known as comb jellies (Fig. 1), are a group of animals found in nearly all marine environments (coastal and oceanic, deep sea as well as surface waters). Most species are planktonic and move by the beating of eight ciliary bands, which is the distinct morphological character uniting the group. About one-third of described species, known as benthic ctenophores, have secondarily derived bilaterally symmetric adult body plans and often live as ectoparasites on other marine invertebrates.

**Fig. 2:**
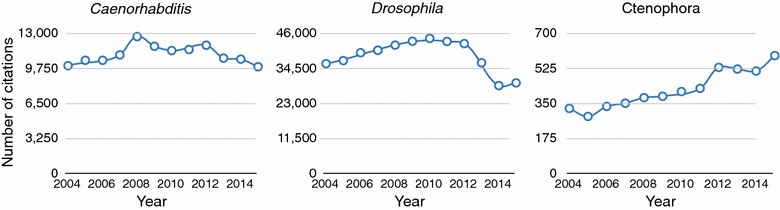
Growing interest in ctenophores is evident in the literature—number of citations from Google Scholar over the past 12 years for the terms “*Caenorhabditis*,” “*Drosophila*,” and “Ctenophora” shows that research on ctenophores is growing. It is interesting to compare both the trends, as well as the enormous difference in numbers of research articles published on these different organisms

The late nineteenth century was a golden age of ctenophore research during which many key discoveries were made (e.g., [1–4]). Yet, these animals went largely unstudied for most of the twentieth century [5]. Several factors have led to a resurgence in interest of ctenophores of late (Fig. 2), including: (1) new ideas about the phylogenetic position of the group [6–11], (2) implications of their phylogenetic position on the evolution of animal cell types [8, 9, 12–17]—but see also [18–20], (3) improved protocols for molecular experimentation [21–27], and (4) new ideas about their ecology and invasive biology [28–33]. In light of this growing interest, we organized Ctenopalooza, the first formal international meeting dedicated to ctenophore biology (ctenophorology).

On March 14–15, 2016, 75 researchers representing 12 countries met for 2 days at the University of Florida’s Whitney Laboratory for Marine Bioscience in St. Augustine, FL, to discuss ctenophorology. There were 50 accepted abstracts, 26 oral presentations, and 24 posters. Below are brief descriptions of the oral presentations (organized chronologically by session) and some of the ideas that surfaced during the meeting. More information including abstracts and videos of many of the talks can be found at the meeting Web site: http://ctenopalooza.whitney.ufl.edu.

## Fossils/diversity/phylogeny

The first presentation of the meeting was a comprehensive review of the ctenophore fossil record by George Stanley from the University of Montana. Stanley gave a detailed assessment of each of the dozen or so relevant published fossils. His take-home message was that interpreting the current ctenophore fossil record is challenging. Stanley called for a revision of the record with special consideration for the effects of taphonomy (the process of fossilization) in evaluating these fossils. Laurence Madin from the Woods Hole Oceanographic Institution presented a history of our understanding of ctenophore diversity. Unlike most animals, ctenophores are very delicate and therefore not amenable to identification when collected with a trawl or net. Madin recounted how before open-ocean SCUBA diving and submersibles there was hardly any information about the deep ocean diversity of ctenophores, and it was thought there were only a limited number of species occupying a very narrow geographical range. These new technologies have completely revolutionized our view of ctenophore biodiversity and have shown that there are upwards of about 200 species widely distributed throughout the Earth’s oceans. Steven Haddock from the Monterey Bay Aquarium Research Institute spoke next about the phylogenetic relationships of ctenophore lineages. He showed photographs of several undescribed ctenophore species that have been collected from the deep ocean. Haddock gave a sneak peak at a taxonomic key that his laboratory is developing for ctenophores and also gave some background on the Diversity, Evolution, and EcoPhysiology of Ctenophores (DEEPC) Project, an effort to understand the evolution and diversification of ctenophores using the deep-sea habitat as a generating force of novel adaptations. The last speaker of the session, Claudia Mills from the University of Washington’s Friday Harbor Labs spoke about a recently discovered deep-sea ctenophore from the northeast Pacific Ocean (species is undescribed at the time of this publication). These exciting ctenophores have lobes, tiny comb rows, and weird knobs. They are transparent in color, but the meridional canals can be strikingly golden in color after ingesting their prey *Poeobius meseres* (Annelida). They use a unique lobe-extension strategy to capture these annelids, which are much larger than their ctenophore predator.

## Nerve and muscle cells

Mari-Luz Hernandez-Nicaise from the University of Nice gave the first keynote of the meeting describing her extensive ultrastructural and cytochemical results on the nervous system of ctenophores and its characteristic synaptic contacts onto sensory receptors and muscle cells—suggesting the presence of cholinergic and aminergic vesicles. The ultrastructure and physiology of *Beroe* giant mesogleal smooth muscles, and the possible mechanisms of calcium activation of muscle contraction without a T-system were discussed. Timothy Jegla from Penn State University spoke about what ctenophores can tell us about the functional evolution of neurites. Jegla discussed the techniques and experiments that are needed to understand the origin of the nervous system, and he put forth the hypothesis that ctenophores branched off prior to the evolution of neural polarity. David Simmons, a Ph.D. student from the University of Florida’s Whitney Laboratory for Marine Bioscience, presented new single-embryo time course RNA-Seq data that compared gene expression patterns during early development in the ctenophores *Mnemiopsis leidyi* and *Beroe ovata*. He showed evidence that many of the genes involved in the bilaterian nervous system (e.g., neurexin, amos, paraxis, shaker channels, and glutamate receptors) show peaks of expression at around 11 h, suggesting that the nervous system is patterned around this time.

## Ecology/behavior/taxonomy

Christian Sardet from Villefranche sur Mer revisited some classic experiments from more than 30 years ago when he and his colleagues revealed an intriguing intracellular behavior of *Beroe ovata* whereby the female nuclei probe different male nuclei before fusing with one [34]. This extraordinary observation has largely remained unstudied since the original paper, but with new tools and better technology now available, seems certain to be revisited. Sardet also shared video footage from the Tara Oceans expedition that took place from 2009 to 2013 [35]. Jamileh Javidpour from the GEOMAR-Helmoltz Centre for Ocean Research in Kiel, Germany, next presented work looking at functional traits in the invasive ctenophore *M. leidyi*. Javidpour presented long-term ecological data showing evidence that cannibalism occurs in *M. leidyi* and that it plays an important role in the population dynamics of *M. leidyi*. She used laboratory experiments to demonstrate that adult *M. leidyi* actively feed on *M. leidyi* larvae. These data offer new insights into the ability of this ctenophore to proliferate and survive in new habitats.

Daniel Sasson, a post-doctoral researcher from the Whitney Laboratory, presented recently published data showing the influence of light, circadian rhythm, and body size on *M. leidyi* spawning [36]. The data challenged some earlier ideas of how light influences spawning and showed results consistent with inbreeding costs associated with self-fertilization. Katharina Bading, a master’s student from GEOMAR, presented evidence that regeneration ability of *M. leidyi* depends on the availability of food. Her results showed that there are energetic limitations to regenerative success in *M. leidyi*, which has implications regarding the invasive abilities of larval *M. leidyi*. Otto Oliveira from the Universidade Federal do ABC in Brazil gave an outline of classification challenges in Ctenophora, pointing out that current classifications at the genera and species level are fairly sound, but that the sub-genera classifications are essentially wrong. Oliveira suggested that a workshop (perhaps at the next Ctenopalooza) be held to sort out the current classification system.

## Metagenomics/slime

Richard Mariita, a Ph.D. student from Auburn University, opened day two of the meeting with a talk about the microbiome of *M. leidyi*. Mariita found that the composition, structure, and functional capabilities of the *M. leidyi* microbiota shifts with the season, but that at least one species of bacteria—*Propionibacterium acnes*—is retained throughout the year. Mya Breitbart from the University of South Florida presented published work on the discovery of novel circular single-stranded DNA viruses in *M. leidyi* [37]. Breitbart reviewed the first reported instances of viruses in ctenophores and also presented preliminary work, suggesting that these viruses may persist year-round. James Townsend, a Ph.D. student from the University of Pennsylvania, presented calorimetric, rheometric, and magnetic resonance imaging (MRI) data with an aim toward identifying physical, biochemical, and anatomic data on the mesoglea of *M. leidyi*. Townsend concluded that *M. leidyi* mesoglea is physiochemically distinct from that of other animal tissues.

## Genes/cells/development

Jason Presnell, a Ph.D. student from the University of Miami, presented an analysis of the Kruppel-like factor (KLF) gene family in *M. leidyi* and *Pleurobrachia bachei*. Presnell showed that in *M. leidyi*, a KLF gene related to KLF5 is expressed ubiquitously early in development and later becomes isolated to endodermal canals. These expression domains are consistent with a conserved cell proliferative role for this gene and warrant follow-up with functional techniques. Richard McCann from Mercer University School of Medicine spoke next about the evolution of the core adhesome (i.e., cell–cell and cell–matrix adhesion receptors). McCann showed phylogenetic evidence for an increase in evolutionary rate of the core adhesome after animals split from the rest of eukaryotes.

William Browne spoke next about the ctenophore through gut. Browne recounted how Chun and others had showed evidence more than 100 years ago that the anal pores of ctenophores were used for excretory functions [38], but that in the modern literature it is thought the majority of digested material is expelled through the mouth [39]. Browne then showed video footage of a ctenophore digesting a fluorescently labeled zebrafish, with high-resolution detail of the ctenophore digestive process leaving no doubt that the vast majority of digested materials exit through the anal pores. Lauren Vandepas, a Ph.D. student from the University of Washington, next presented successful isolation and maintenance of multiple cell types from primary cultures of *M. leidyi*. The availability of ctenophore cell lines will enhance cellular level research on ctenophores.

## Evolutionary genomics

Nathan Whelan, a post-doctoral researcher from Auburn University, presented phylogenetic relationships of ctenophore lineages. Whelan used Bayesian ancestral state reconstruction to infer that the last common ctenophore ancestor had a cydippid body plan and had a pelagic lifestyle. Christine Schnitzler from the National Human Genome Research Institute gave an overview of the completeness of two publicly available ctenophore datasets (*M. leidyi* and *P. bachei*) as well as a third unpublished genome sequence (*Beroe ovata*). Schnitzler’s measures of completeness (including comparisons to a sponge genome) are important for making inferences about gene structure and loss when using these data. Andrea Kohn from the Whitney Laboratory spoke about the epigenetic landscape in ctenophores. Kohn showed that DNA demethylation is prominent during development in *M. leidyi*, *P. bachei*, and *Beroe abyssicola* [40]. Kohn also showed evidence for expansion of the RNA-editing machinery in ctenophores.

## Benthic ctenophores

There was an exciting session on an enigmatic group of ctenophores called platyctenes or benthic ctenophores. Peter Glynn from University of Miami’s Rosenstiel School of Marine and Atmospheric Science spoke about benthic ctenophores in South Florida. Glynn discussed the habitat, population dynamics, feeding and reproductive behavior, predators, and ecological roles of the benthic ctenophores *Coeloplana waltoni* and *Vallicula multiformis*. He showed that the rate of Coeloplana species discoveries is linear, which implies that there are many more Coeloplana to be discovered. George Matsumoto from the Monterey Bay Aquarium Research Institute spoke about the taxonomy of benthic ctenophores. Matsumoto went through each family (Savangiidae, Lyrocteidae, Coeloplana, Tjalfiellidae, and Ctenoplanidae) including a new undescribed family that appears to have only four comb rows and was discovered at a depth of 7000 meters (thought to be the greatest depth that a ctenophore has been found to date). Gustav Paulay from the University of Florida’s Museum of Natural History spoke about benthic ctenophore diversity in the Indo-West Pacific. Paulay began by pointing out that the vast majority of the known marine biodiversity is benthic (~23X), but according to the World Register of Marine Species (WoRMS) [41] there are 169 pelagic ctenophores and 59 benthic species, suggesting that there are many more benthic species to be identified. He then shared photographs from many benthic ctenophores that co-occur on echinoderms, soft corals, and algae with special emphasis on host specificity and the occurrence of several species on single hosts (e.g., eight species on toadstool coral *Sarcophyton*). Paulay discussed *Coeloplana meteoris*, a ctenophore that spends most of its time on the benthos, but unlike most benthic ctenophores, has maintained its ability to swim. These ctenophores have an oral groove that extends to the tentacles, which is characteristic of *Vallicula*. This is interesting given their three-dimensional structure (unlike other Coeloplanids) and phylogenetic data (presented earlier by Whelan), showing that *C. meteoris* are more closely related to *Vallicula* than to other coeloplanids. Paulay suggests that they should be placed in their own genus *Benthoplana*. Lastly, he described *Lobatolampea tetragona*—a lobate that lives on soft bottoms but can lift and swim. These animals have very distinct morphology including tentacles that extend around its lobe, and unlike most other ctenophores, they brood their embryos.

## Closing keynote

Casey Dunn from Brown University gave the closing talk—the second keynote—entitled Ten-Four Ctenophore. Dunn reviewed the current literature surrounding the position of Ctenophora on the animal tree of life. He showed that the support for Porifera as the sister group is highly dependent on using only Choanoflagellata as an outgroup and only with the CAT + GTR framework. He argued that these conditions as well as the argument that all other approaches lack explicit criteria other than tree topology are not justified. Dunn pointed out that ctenophores have a great deal to offer as to how we interpret variation in biology and how we understand animal diversity. Along these lines, he had the following calls to action (1) stop shoehorning ctenophore biology into cnidarian and bilaterian biology, (2) better communicate why ctenophores are interesting with an emphasis on how they differ from other animals, (3) meet again in the next few years (“because this has been fun and interesting”), and (4) seek out the unique biology of ctenophores (e.g., "let’s have five whole talks devoted to colloblasts”). He closed his talk with a tongue-in-cheek apology to ctenophores, promising that we will do a better job of celebrating their uniqueness.

## Poster sessions

Two poster sessions were each opened with 2-min lightning talks where each presenter gave an overview of their poster. These lightning talks successfully sparked discussion among participants and led to lively discourse in front of posters. The posters featured a range of research topics including biomechanics, biogeography, regeneration, neuroscience, embryology, genomics, invasive biology, horizontal gene transfer and more. Two posters even included tanks with live benthic ctenophores!

## The future of ctenophore research

The future of ctenophorology is bright. A discussion during the last session of the meeting led to ideas on important topics for future research centering around the remarkable biology of these animals. Some examples of topics put forward were: bioluminescence, macrocilia (e.g., the “teeth” of *Beroe*), colloblasts, the statolith, unipolar cleavage, male/female pronuclear fusion, non-polarized neurites, a Hox-free primary body axis, and invasive biology. It will be important to establish a powerful experimental model ctenophore that can be used to address many of these topics. *M. leidyi* is the most obvious choice given the accessibility of these animals, as well as the availability of a high-quality genome and established experimental techniques. The implementation of these techniques in other ctenophores should also not be far off and will be important for addressing comparative questions. The establishment of new experimental techniques in ctenophores combined with the influx of new ctenophorologists will lead to a greater understanding of both the unique and shared characteristics of these fascinating animals. We look forward to witnessing first hand the growth of ctenophore biology and comparing the scope and magnitude of this inaugural meeting to future Ctenopaloozas.

## Abbreviations

DEEPC: Diversity, Evolution, and EcoPhysiology of Ctenophores
KLF: Kruppel-like factor
MRI: Magnetic resonance imaging
WoRMS: World Register of Marine Species
